# Sociodemographic and Population Exposure to Upstream Oil and Gas Operations in Canada

**DOI:** 10.3390/ijerph21121692

**Published:** 2024-12-19

**Authors:** Martin Lavoie, David Risk, Daniel Rainham

**Affiliations:** 1 Department of Earth and Environmental Sciences, St. Francis Xavier University, Antigonish, NS B2G 2W5, Canada; drisk@stfx.ca; 2 School of Health and Human Performance, Dalhousie University, Halifax, NS B3H 4R2, Canada; daniel.rainham@dal.ca; 3 Healthy Populations Institute, Dalhousie University, Halifax, NS B3H 4R2, Canada

**Keywords:** methane, nitrous oxide, air pollutants, air quality, oil and gas, CanCHEC, DAD

## Abstract

Canada, as one of the largest oil and gas producer in the world, is responsible for large emissions of methane, a powerful greenhouse gas. At low levels, methane is not a direct threat to human health; however, human health is affected by exposure to pollutants co-emitted with methane. The objectives of this research were to estimate and map pollutants emitted by the oil and gas industry, to assess the demographic of the population exposed to oil and gas activities, and to characterize the impact of well density on cardiovascular- and respiratory-related outcomes with a focus on Alberta. We estimated that ~13% and 3% people in Alberta reside, respectively, within 1.5 km of an active well and 1.5 km of a flare. Our analysis suggests that racial and socioeconomic disparities exist in residential proximity to active wells, with people of Aboriginal identity and people with less education being more exposed to active wells than the general population. We found increased odds of cardiovascular-related (1.13–1.29 for low active well density) and respiratory-related (1.07–1.19 for low active well density) outcomes with exposure to wells. Close to 100 countries produce oil and gas, making this a global issue. There is an important need for additional studies from other producing jurisdictions outside the United States.

## 1. Introduction

At sites of oil and gas production, methane (CH_4_) is emitted during natural gas flaring, venting, and fugitive leakage, as well as from combustion, storage, and handling losses. Methane is eighty-two times more potent on a 20-year timescale than carbon dioxide (CO_2_) as a greenhouse gas with adverse climate effects [1]. Although exposure to methane can be a threat at higher concentrations, such asphyxiation in work settings, the principal health concern is exposure to other pollutants co-emitted with methane including volatile organic compounds (VOCs) and other airborne toxicants [2,3]. In addition to direct toxicity, pollutants such as ozone can form as a by-product of methane and VOC emissions when they react with sunlight. The International Energy Agency (IEA) has listed more than 50 countries supplying more than 100,000 TJ/year of crude oil or natural gas. This means that many people, potentially up to 600 million, may be exposed to harmful pollutants produced by the oil and gas industry [4].

Most studies looking at the impact of oil and gas activities on human health outcomes originate from the United States, the largest producing country in the world [5]. Approximately 20% of global oil and 25% of natural gas supplies are produced by the United States. Several past studies found that air pollutant concentrations and associated health risks (e.g., wheezing, eye and nose irritation, sore throat, dizziness, cardiovascular and respiratory disease, excess death) increased with proximity to an oil and gas facility [6,7,8,9,10]. We also know from other U.S. studies that flaring results in the release of harmful compounds, including particulate matter (PM_2.5_), nitrogen oxides (NO_x_), sulfur compounds, and unburnt VOCs [11,12]. Known health effects from flaring include increased incidence of preterm birth [13], asthma, pulmonary problems, nervous disorders, mental health conditions, and reductions in overall life expectancy [14,15,16].

In second-tier producing countries like Canada (fourth largest globally), we have seen comparatively few studies on the association between exposure to oil and gas activities and human health impacts [17]. Well densities are up to 3 times lower in Canada than they are in dense producing regions of the United States, implying that air quality impacts like respiratory and cardiovascular health problems should be more limited there, as in other second-tier producing countries. Given the availability of oil and gas and health datasets, Canada is a good test case for assessing the environmental health impacts of oil and gas in other second-tier oil and gas-producing nations. In addition, Canada has similar oil and gas practices to the US, so differences in health outcomes would be mainly a function of production infrastructure density.

In Canada, we have early research examples of air impacts on cattle (rather than human) health [18,19]. In the province of British Columbia, research suggests that well density/proximity has been associated with increased human exposure to certain VOCs [20,21] and fine particulate matter (PM_2.5_) [22]. These results suggested that airway transmission was the main pathway of exposure. For example, Caron-Beaudoin et al. [20] identified 40 different VOCs in >50% of air samples, whereas only 4 VOCs were identified in >50% of water samples. Another study in northeastern British Columbia also showed some evidence of greater odds of mental illness with the presence of oil and gas extraction wells, but no association was found at the closest distance [23]. Based on our systematic review of the literature, only two studies have investigated the role and impact of oil and gas activities on human health in the largest oil-producing province of Canada: Alberta. The results of the first study suggest that exposure (within 10 km) to hydraulic fracturing during preconception or pregnancy increases the risk of several adverse birth outcomes, including small for gestational age, major congenital anomalies, and spontaneous preterm birth [24]. In the second study, the authors reported a significant correlation between oil and gas infrastructure and solid tumour incidence [25]. However, in both studies, it was not investigated by which specific mechanism (e.g., air, water) oil and gas activities had an impact on health outcomes. In a recent study in northeastern British Columbia, indigenous pregnant women had higher median concentrations of barium (2 times), cobalt (3 times), and strontium (2 times) in their urine than non-indigenous pregnant women [26]. Despite these studies, there is an absence of research in Canada on the impacts of oil and gas-derived air pollution on respiratory and cardiovascular health problems. Similarly, the sociodemographic portrait of the population exposed to oil and gas in Canada is also poorly characterized [27]. This is a topic that is comparatively well understood in the United States, where 17.6 million people live within 1.6 km of at least one active oil and/or gas well [28], and of the 0.5 million people who reside within 5 km of a flare in major basins, poor and that disadvantaged populations are disproportionately exposed [29,30], especially Black and Hispanic residents in the states of Texas and California [31]. For second-tier producing countries like Canada, we know less about exposed populations.

For this multidisciplinary study, we must work with many different datasets to understand exposure and health outcomes related to oil and gas. Specifically, we leverage multiple industry and government databases to build a sector-specific spatial exposure model of atmospheric pollution from oil and gas production in Canada. Using ground and satellite observational data, we then validate the spatial model and use it to establish pollution levels exposure in urban and rural areas, as well as among different demographic communities identifiable from census databases. Finally, we use a federal health database to test whether the respiratory or cardiovascular health outcomes of these communities are negatively influenced by exposure levels. While such databases may not be accessible in all countries, this analysis is likely broadly representative of outcomes in other well-regulated, midsized oil and gas-producing nations, where similar research gaps may also be present.

In this study, we test two hypotheses. Considering that several studies have shown a negative correlation between distance and the prevalence of health symptoms [7,32,33], we first hypothesize that rural and marginalized communities will be at the greatest risk of exposure to air pollution from oil and gas development and that urban dwellers will be far less affected by pollution from the sector. Second, considering that there is link between well density and health outcomes [34], we hypothesize that even in some less populated rural areas, some demographics and communities will experience diminished health outcomes from nearby oil and gas development, but that severities may scale with infrastructural density.

## 2. Materials and Methods

### 2.1. Study Area

Over 80% of Canada’s oil production comes from the province of Alberta. Alberta also produced 61% of Canada’s total natural gas production in 2023. Our inventory for the study period included 157,339 (range = 146,330–167,259) oil and gas wells and 21,385 (range = 18,574–23,927) facilities that were active at some point during years 2016–2021 (Tables S1 and S2). Annual production volumes averaged 1.5 billion barrels of oil equivalent (BOE), excluding mined oil sands.

### 2.2. Inventory Methods

**Figure 1:**
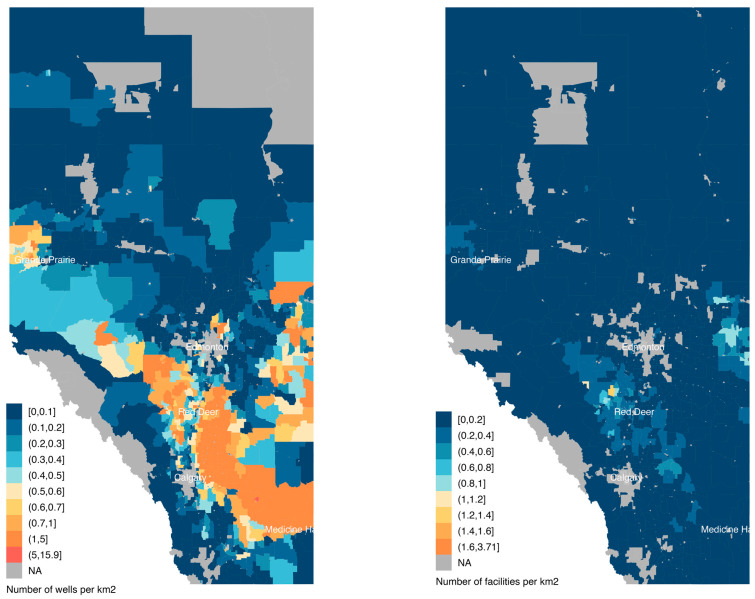
Number of active wells and facilities per square kilometer during the years 2016–2021 in Alberta, Canada. NA = No data reported or no wells present.

For this study, we used data provided by the Petrinex production accounting system [35] used by provincial Regulators, including the Alberta Energy Regulator (AER), the British Columbia Energy Regulator (BCER), and the Saskatchewan Ministry of Energy and Resources (MER). Active facilities and wells were defined as any site that reported produced oil or gas volumes; flared, vented, or natural gas fuel volumes; or any gas dispositions or receipts. We selected the years 2016–2021 based on data availability, and the spatial distributions of facilities and wells are shown in Figure 1 and Figure S1. We used geolocations of facilities and wells provided by the database service provider IHS (https://ihsmarkit.com, accessed on 10 July 2023) to locate oil and gas infrastructure.

Pollutants are emitted by flaring, venting, onsite fuel combustion, and fugitive emissions. Oil and gas production and flare and onsite fuel combustion volumes were extracted using the Petrinex database. Fugitive emissions were calculated using emission factors from the 2022 Environment Climate Change Canada (ECCC) National Inventory Report [36], as submitted to the United Nations Framework Convention on Climate Change (UNFCCC; see the Supplementary File S1 “Activity and CH_4_ Emission Factors”).

Recent studies have shown that reported fugitive emissions in Canada during the study years were underestimated by a factor of 1.5–2 [37,38]. Recent changes to the National Inventory Report, subsequent to our chosen study period, reset oil and gas methane emission levels and rectified the inventory gap that arose largely from regions producing cold heavy oil (i.e., Cold Heavy Oil production with Sand (CHOPS)) near Lloydminster, Alberta–Saskatchewan [39,40]. In the CHOPS region, emissions were up to 5x higher than reported because of uncertainties in the gas/oil ratio (GOR) and other factors. For the study period, however, we needed to update the inventory values, and we used a correction factor to calculate more accurately venting emissions coming out of CHOPS sites. The excess emissions were calculated by applying an additional 56 kg of CH_4_ per m^3^ of oil produced by each CHOPS site [41]. A recent analysis has also suggested that abandoned well emissions in Canada are 2.5 times higher than currently estimated [42]. Inactive wells (suspended and abandoned) are also known to emit methane and other pollutants [43,44]. To correct for that, we multiplied the emission factors for abandoned wells by 2.5.

Pollutant emission estimates for VOCs, NO_X_, and PM_2.5_ were calculated from reported gas emissions (as calculated in Section 2.2), flaring, and onsite fuel combustion volumes using emissions factors provided by ECCC (see Supplementary File S2 “Pollutant Inventory equations”).Additional details on conversion factors can be found in Canada’s Air Pollutant Emissions Inventory Report—2022 Edition (Environment and Climate Change Canada, 2023) [45].

To be able to assess the impacts of oil and gas activities on cardiovascular and respiratory problems (see Section 2.3), we aggregated the variables of interest (e.g., pollutant emissions, well density, etc.) to the dissemination area (DA) scale. The dissemination area is the smallest standard geographic area for which all Canadian census data (e.g., population, age, income, etc.) are disseminated [46]. Dissemination areas vary in population size (300–700 people), and area with larger DAs located in rural areas and higher numbers of DAs were found in major cities.

### 2.3. Exposure Assessment

The proportion of people living near (<1 km and <1.5 km) an active oil or gas or exposed to flare was calculated with the simplifying assumption that residents were evenly distributed throughout the DAs [31]. We assessed the proportion of each DA that intersected with 1 km and 1.5 km buffers around active oil and gas wells and 1.5 km buffers around flares. The distances of 1 and 1.5 km were selected based on previous results from the literature [47,48].

For this analysis, we included race/ethnicity, education, renter-occupied household, income, and age. Using data from the 2016 Census [49], we calculated risk ratios (rr) to determine whether marginalized people disproportionately resided near active wells (within 1.5 km). We defined the risk ratios as the proportion of individuals in a specific demographic group who were exposed to the proportion of the total population who were exposed [31]. To calculate the number of residents located in proximity of well, we assumed that residents were evenly distributed across the dissemination area. A risk ratio greater than 1 indicated that the group was over-represented among the exposed population. Equally, groups that were under-represented among the exposed population would have a risk ratio less than 1. Groups that were exposed in proportion to that group’s total population would have a risk ratio near or equal to 1.

To test for the presence of health impacts, we employed a logistic regression model to estimate adjusted odds ratios (ORs) [50] for the association between exposure to well site density (active, inactive), TROPOMI NO_2_ concentration (see Section 2.5), and the outcomes of interest (e.g., cardiovascular- and respiratory-related outcomes). We also added sex, age, location (urban/rural), and income (Neighbourhood Income Quintile Before Tax) into the adjusted model. An odds ratio of 1.06 would indicate that there is a 6% increase in the odds of cardiovascular outcomes with a given exposure to well density or the presence or absence of wells.

We used the Canadian Census Health and Environment Cohorts (CanCHEC) and Discharge Abstract Database (DAD) developed by Statistics Canada [51] and the Canadian Institute for Health Information [52]. The DAD contains administrative, clinical, and demographic information on hospital discharges (including in-hospital deaths, sign-outs, and transfers) from all provinces and territories, except Quebec. We included the years 2012–2016 to cover the period between the 2016 and 2011 Census datasets (the 2021 Census was not available yet). The DAD has been used in previous studies, including a recent study of the impact of ambient air pollution on the incidence of acute myocardial [53] and Alzheimer’s disease [54]. ICD-10 hospitalizations related to cardiovascular and respiratory related outcomes were defined using codes I00 to I99 and J00 to J99, respectively [55].

### 2.4. ECCC National Air Pollution Surveillance Program

To investigate the relationship between population size and pollutant air concentration, we used the publicly available long-term air pollutant data from the ECCC—National Air Pollution Surveillance Program (Environment and Climate Change Canada, 2010) [56]. We extracted, for all the monitoring stations, the 2021 NO_x_ concentration data and then computed the annual NO_x_ concentration for each station. Air pollutants monitored continuously by the ECCC stations included the following pollutants: carbon monoxide, nitrogen dioxide, nitric oxide, nitrogen oxides, ozone, sulfur dioxide, and particulate matter (PM_2.5_, PM_10_). For the analysis, we selected NO_x_ since transportation, flaring, and onsite fuel combustion are the main contributors to total NO_x_ emissions in rural areas (Environment and Climate Change Canada, 2023) [57].

### 2.5. Satellite Remote Sensing Dataset

In addition to NO_x_ emissions from oil and gas activities, we included TROPOMI NO_2_ concentrations as a predictor in our logistic regression model. In the United States, a study showed that TROPOMI had a very good capability for observing the spatial patterns of NO_2_ pollution [58].There was also a strong relationship between mean annual NOx (ppb) measured at airshed field monitoring stations (see Section 2.6) and TROPOMI tropospheric vertical column densities of NO_2_ in Alberta during 2021 (r = 0.74; *p* < 0.0001; Figure S5). Satellite-retrieved NO_2_ total column densities were from the TROPOMI instrument, which has been flown on the European Space Agency (ESA) Sentinel-5p satellite since 2017 and has provided observations since mid-2018. TROPOMI is a spectrometer measuring solar backscatter radiation in the ultraviolet–visible (UV–vis) spectral bands. TROPOMI provides daily, global observations on sun-synchronous orbits with a local overpass time around 1:30 p.m. We used the level 2 version 2 reprocessed (RPRO) dataset. The resolution of the TROPOMI NO_2_ pixels was ~3.5 × 5.5 km^2^; however, we used a physical oversampling algorithm [59] to aggregate the daily NO_2_ retrievals to monthly and annual grids with a pixel resolution of 1 × 1 km^2^, consistent with several previous studies [60,61,62].

In this study, TROPOMI tropospheric vertical column densities of NO_2_ for 2021 were used as proxies and may not have been representative of the actual NO_2_ concentrations for the period of interest (2011–2016). Due to emission reduction policies, NO_2_ concentrations measured across Alberta were generally lower in 2021 than between 2011 and 2016 [63]. However, considering that roughly 40% and 60% of the NO_2_ emissions, respectively, came from transportation and oil and gas, it is likely that the spatial distribution of the NO_2_ concentration was similar during those years.

### 2.6. Airshed Ground Monitoring Data

Ground monitoring data were provided by Airsheds in Alberta [64]. Airsheds now operate more than 88 air monitoring stations across Alberta in compliance with all provincial and federal standards (Figure S6). Data (NO_x_ annual concentration) from seventy stations were used in this study. The data are publicly available on the Alberta Air Data Warehouse.

### 2.7. Review of Literature

To gather data and information on our topic of research, we also performed an extensive review of the literature by searching for previous peer-reviewed and non-peer-reviewed studies by using Google, Google Scholar, ISI Web of Science, MEDLINE, journal publishers (e.g., Elsevier, MDPI, Springer, Taylor, Wiley, etc.), EarthXiv, and ChemRxiv databases. Relevant articles were found using keywords, in various combinations, such as oil and gas production, methane emissions, nitrogen oxides (NO_x_), fine particulate matter (PM_2.5_), VOCs, respiratory outcomes/health, cardiovascular outcomes/health, and Canada/Alberta. Relevant articles were then used to identify other relevant sources by consulting the ‘cited by’ list. After the initial search, alerts (Google, journal publishers) were created to allow us to be informed of the latest research. The search was not limited to Canada and the U.S. only, but most of the studies were from the US, and only a few were from Canada.

## 3. Results and Discussion

### 3.1. Oil and Gas Activities

Annual average flared volumes from all upstream oil and gas sources were 800 × 10^6^ m^3^ yr^−1^ on average for Alberta (2016–2021). The total volume vented from all upstream oil and gas sources was 233 × 10^6^ m^3^ yr^−1^, and the average annual volume gas used as onsite fuel was 23,900 × 10^6^ m^3^ yr^−1^. The trend during the study period was relatively stable for onsite fuel, but an increasing trend was noted for vented and flared volumes in part due to a change in definition in the regulations [65].

Pollutants are not only emitted when venting or flaring but also with fugitive emissions. For Alberta, fugitive methane emissions were 4,556,715 m^3^ per day (~1243 kt per year) on average during the study period. This value was significantly higher than the one (987 kt or 3,618,368 m^3^ per day for 2021) reported in the 2021 National Inventory Report [66] but is in line with the new 2024 National Inventory Report [67] released recently because the new inventory has repaired emission estimates from CHOPs sites and abandoned wells.

We know that, in Canada, the oil and gas industry is a large contributor to methane emissions, but less is known about how Canada compares to other producing countries for fugitive emissions, venting, and onsite fuel volumes because data are often aggregated and, for most countries, national inventories are based on emissions factors. However, based on this research [68] using VIIRS nighttime observations, Canada was listed among the top 25 flaring countries during the study period.

### 3.2. Pollutant Emissions

The Canadian Council of Ministers of the Environment developed the Canadian Ambient Air Quality Standards for PM_2.5_, O_3_, SO_2_, and NO_2_. The annual air quality criteria set for 2020 were 17 ppb and 8.8 μg/m^3^ respectively for NO_2_ and PM_2.5_. For the same pollutants, the air quality guidelines from the World Health Organization (2021) are 10 μg/m^3^ and 5 μg/m^3^. Larger concentrations are allowed for 1-h, 8-h and 24-h maximum peaks. Air quality criteria or guidelines have not been established for VOCs even though VOCs contribute to the formation of ground-level ozone and PM_2.5_.

National emissions of VOCs, PM_2.5_, and NO_x_ are only reported by province and sector, and not by type of activity (e.g., flaring, fugitive, fuel, losses, etc.). Therefore, we used the national estimates in the 2022 Canada’s Air Pollutant Emissions Inventory Report only to compare total estimates. We also referred to estimated annual emissions reported and published by Clearstone [69]. To the best of our knowledge, this is the only study reporting emissions by activity type. The latest year available estimated in this report was for 2000. We expect our estimates to be lower than those for the last year (2000) reported by Clearstone since flaring and venting activities and associated emissions have declined over time [70,71]. As a reminder, our estimates from the upstream oil and gas do not include emissions from the mined oil sands in northeastern Alberta.

**Figure 2:**
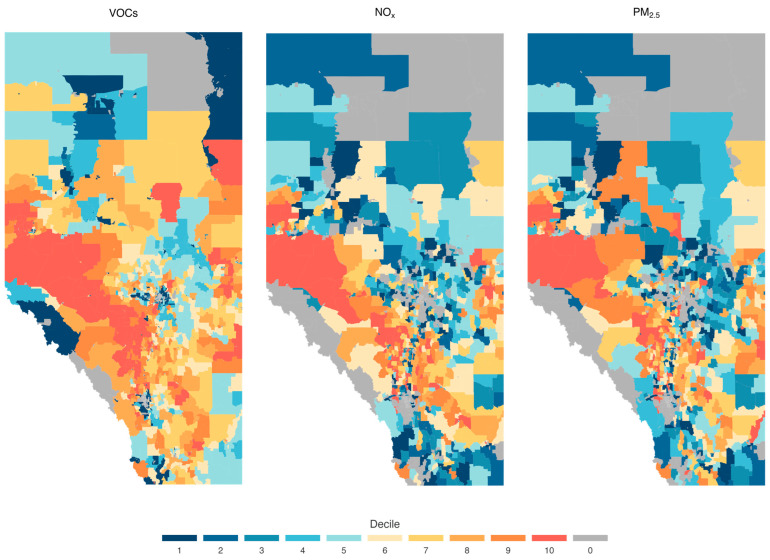
Estimated PM_2.5_ density per DA, NOx density per DA, and VOCs density per DA for Alberta, Canada, during the years 2016–2021. The emission units for the three pollutants are initially kg per km^2^ per year. Then, the DAs with emissions estimates are divided in 10 equal parts (deciles). Thus, each decile has the same number of DAs. DAs with zero (gray) indicates that no emissions were reported.

Figure 2 shows the spatial distribution of the three selected pollutant emissions. In this figure, the emission units for the three pollutants shown are kg per km^2^ per year. However, for better visualization, the DAs with emissions estimates (i.e., excluding DAs with no emissions) were divided in 10 equal parts (deciles). Flaring, venting, onsite fuel use, and oil and gas activities are generally spread out across the province of Alberta. However, the highest concentrations of pollutant emissions were generally found along the corridor (between Edmonton and Calgary) from Medicine Hat in the southeast to Grande Prairie in the northwest. Emissions were also higher near the Lloydminster–Bonnyville area in central-eastern Alberta, where CHOPS production is important.

Overall, the annual average of PM_2.5_ emissions due to flaring was 1800 t/y (metric tonne per year). For onsite fuel use, the annual average was 1200 t/y. Our PM_2.5_ emission estimation of 3000 t/y is in line with estimates from Clearstone [69], with PM_2.5_ emissions due to flaring and combustion being estimated at 6368 t/y when flaring occurred twice as much as today. The total upstream oil and gas sector emissions for the country (with Alberta being the largest contributor) were 11,000 t/y, which was less than 1% of the total PM_2.5_ emissions emitted in 2020. In comparison, at 3000 t/y for the country, it is significantly less than the 25,000 t/y reported for the United States [6].

NO_x_ emissions from flaring and onsite fuel use were on average 950 t/y and 257,971 t/y, which were lower than those reported in the 2020 National Air Pollutant Inventory but within the range of those (2024/310,514 t/y for year 2000) reported in the Clearstone report [69]. In 2021, Alberta emitted the most NO_x_—40%, or 530 kt—of all Canadian provinces and territories. The oil and gas industry was an important source of NO_x_ emissions in Alberta, accounting for 65% (344 kt) [57] of total emissions, with transportation emissions being the other major source. In comparison, the contribution of the oil and gas industry to NO_x_ emissions in the United States was estimated to be about 1,000,000 t/y [6,72], which is in line with the contributions of the United States (~17%) and Canada (~6%) to global oil production.

Alberta was also the highest emitter of VOCs in the country. Using production data and emission factors, we estimated 58,732 t/y and 3785 t/y of VOCs emitted, respectively, from fugitive emissions and onsite fuel use in Alberta. In comparison, the total annual estimated emissions of VOCs for Canada in 2020 were 510,000 t/y, and Alberta represented roughly 33% (464,000 t) of national emissions. The Clearstone report [69] suggested that roughly 26% of VOC emissions were attributed to fugitive and onsite fuel use venting emissions (i.e., ~123,000 t/y for AB). VOCs are also emitted substantially from surface casing vent flow/gas migration, glycol dehydrator, loading/unloading, spills, and storage losses. VOC emissions from oil and gas are an important contributor (~30%) to total VOC emissions [57]. In the same U.S. studies cited above [6,72], it was also reported that the air pollution contribution of oil and gas activities to VOC emissions was more than 3,000,000 t/y, about 6–7x more than was reported for Canada.

A comparison of study estimates with official reported values is challenging. ECCC only publishes inventories by province and broad sectors, not by source type. The only point of comparison was a 2014 report with 2000 as the latest year of reference [69]. Better data availability and more clarity on the emission factors used for each pollutant, sector, and source would be beneficial to estimate and map population exposure to these pollutants. Similarly, a comparison with other jurisdictions and countries with similar levels of production to Canada was also challenging because the majority of the studies are limited to the United States [6,73]. However, satellite-based measurements combined with modelling will improve the quantification of the contribution of oil and gas activities to overall pollutant emissions. They will also facilitate tracking the progress and impacts of new regulations regarding air quality. Satellites are now used to measure several pollutants, including CH_4_ [74], NO_x_ [58,73], PM_2.5_ [75], and VOCs [76].

### 3.3. Proximity to Active Wells

**Figure 3:**
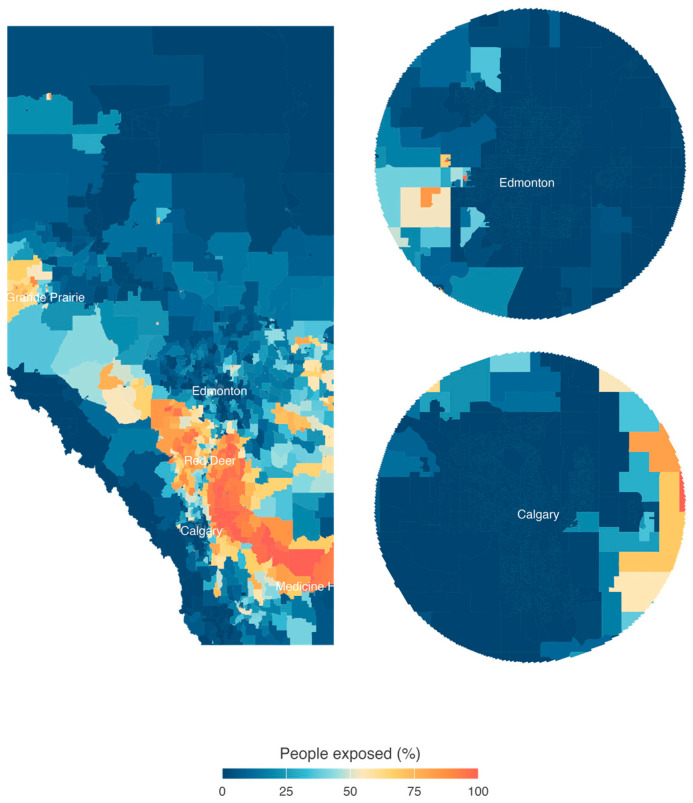
The estimated proportion of people per dissemination area living near (<1 km) an active oil or gas well during the study period in Alberta, Canada, during the years 2016–2021. A zoomed-in (25-km radius) picture of the two largest cities of Alberta: Edmonton and Calgary.

Figure 3 shows the estimated proportion of people per dissemination area living near (<1 km) an active oil or gas well. For the study period, we estimated for Alberta that approximately 360,572 (8.9%) and 546,663 (13.5%) people, respectively, lived within 1 km and 1.5 km of an active oil or gas well. Most people living near a well were found to be living between Medicine Hat (southeastern Alberta) and Red Deer (south-central Alberta). People living near Grande Prairie (western Alberta) and Lloydminster (central-eastern Alberta), two areas with CHOPS production, were also generally living close to an active well. In comparison, ref. [31] found that 3% of Californians lived within 1 km of active wells. Roughly 6% of the conterminous U.S. population was considered to be living within 1.6 km of active wells [28].

We also estimated that approximately 3% (120,000) of the population in Alberta lived within of 1.5 km of a flare. Thus, our results suggest that people in Alberta live in less close proximity to flares than some populations in the United States. A recent study of three major United States basins (Permian, Western Gulf, and Williston, ND, USA) revealed that between 9 and 30% of the population was exposed (<3 km) to flaring [29].

### 3.4. Population Exposure

In larger population centres, such as Calgary and Edmonton, Alberta’s two largest cities, population exposure to air emissions is primarily from transportation-related sources and much less so from oil and gas activities. However, in populations in rural areas, oil and gas is an important source of pollutant emissions. Figure S3 shows, for some towns and cities (excluding Calgary and Edmonton, where there are no oil and gas wells inside the city limits, and populations under 500 consisting mainly of summer villages) in Alberta, the relationship between pollutant emissions (NO_x_, VOCs) and the proportion of people living within 1.5 km of an active well. Population exposure to pollutant emissions is quite variable. Towns with the largest percentage of the population experiencing the highest pollutant load from oil and gas include Drayton Valley, Brooks, Medicine Hat, Rocky Mountain House, and Wembley. We also estimated that at least 5% of municipalities have 50% or more of their population exposed to an estimated 1 tonne (metric) of VOCs km^−2^ yr^−1^ and 1 tonne of NOx km^−2^ yr^−1^ originating from oil and gas operation. In fact, based on NO_x_ concentrations measured by the ECCC National Air Pollution Surveillance Program, our analysis shows that Alberta has significantly (Ancova; *p*-value < 0.0001; no difference in slope) higher concentrations of NO_x_ than any other provinces in Canada (Figure S4). For example, some small towns in Alberta such as Grande Prairie or Drayton Valley showed NO_x_ annual mean concentrations similar to or higher than larger cities like Thunder Bay, Ontario, or Terrebonne, Québec. It is very likely that the pattern that we observed for Alberta would be similar in other countries with oil and gas development and similar levels of production. Fifty percent of the population in Alberta is found in only two cities, and production is mostly concentrated near smaller rural towns and is spread out across the province. In contrast, population exposure in the United States can be quite high in some areas due to high well density in some of the states.

### 3.5. Exposure Inequality

According to the 2016 Census, Alberta was home to 4,067,040 people and had 5803 DAs (mean = 110.28 km^2^; median = 0.28 km^2^). Population density varied significantly (0–73,167 people per km^2^; mean = 2514 people per km^2^; median = 2221 people per km^2^), with the larger urban centres (e.g., Edmonton, Calgary) having the highest population density (Figure S2). Due to the size of their populations, close to 50% of the DAs in Alberta were in the city limits of Calgary and Edmonton. Dissemination areas also vary in size, with larger DAs found in rural areas (40,000–148,900 km^2^) and smaller DAs located in larger cities (0.002–0.006 km^2^).

The percentage of residents with an Aboriginal identity was approximately 6%, while the percentage of the population identifying as a visible minority was 23.5%, with Asian being the most frequent (52%; Table 1). The proportion of people with no diploma was 16.9%, while 65% of residents had an annual income of less than USD 60,000. In addition, the proportion of people over 65 years old was slightly above 12%.

Our analysis suggests racial and socioeconomic disparities in residential proximity to active wells. First, people of Aboriginal identity (risk ratio (rr) = 1.21) and people with no diploma (rr = 1.20) in Alberta were more exposed to active wells than the total population. In contrast, people from visible minorities (rr = 0.36) and renters (rr = 0.75) were less exposed than the total population probably because they live mostly in the largest cities (e.g., Edmonton, Calgary, AB, Canada), where there is no or little upstream oil and gas infrastructure.

In the United States, several studies have shown that marginalized communities, especially Black, Indigenous and Latino communities, are more exposed to oil and gas infrastructure than the general population [29,31]. In Canada, the picture is slightly different. People from visible minorities are highly concentrated in larger cities such as Edmonton and Calgary. High densities of production infrastructure are generally located in rural areas coexisting with agriculture. People exposed to oil and gas wells are more likely to be farmers, rural citizens, and members of Indigenous populations. Our observations concurred with the results published in the 2010 report Rural Alberta Profile—A Fifteen-year census Analysis [77] stating that (1) the proportion of the population that was Indigenous was higher in rural Alberta than in urban regions (11.9% vs. 4.1% in 2006), (2) the proportion of immigrants was lower in rural Alberta than in urban regions (6% vs. 19% in 2006), and (3) the percentage of people with less than a high school education was higher in rural Alberta than in urban regions (33.5% vs. 20.8% in 2006), while the percentage of people with any post-secondary diploma was higher in urban than rural regions (52.9% vs. 40.4% in 2006).

The results of this study highlight the importance of examining further if racially and socioeconomically marginalized people are more exposed to oil and gas activities than the rest of the population. This study showed that it is wrong to base assumptions on studies from different countries and to assume that the same group of people will be necessarily overly exposed. Thus, there is a need for more regional studies that can better picture the population exposed to air pollution.

### 3.6. Effects of Oil and Gas Activities on Cardio-Respiratory Outcomes

**Figure 4:**
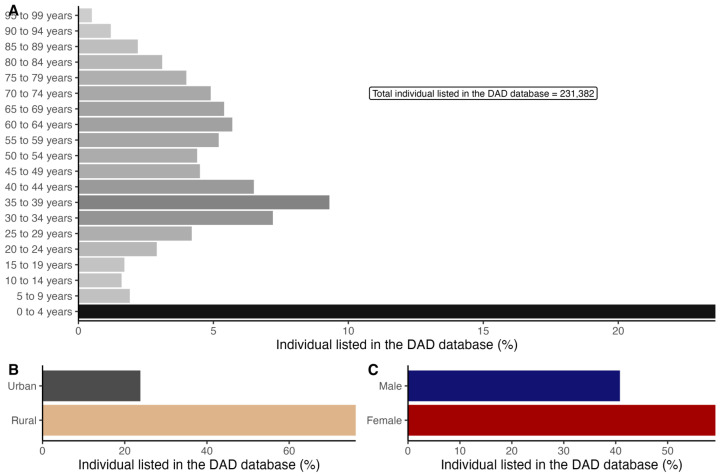
The summary statistics for individuals listed in the DAD database by age category (**A**), location (**B**), and sex (**C**). Urban/rural is defined using the Statistical Area Classification type (SACtype). For this study, SACtype descriptions 1 (census subdivision with census metropolitan area), 2, and 3 are designated as urban and categories 4 to 8 (census subdivision withing territories, outside of census agglomeration) as rural.

**Figure 5:**
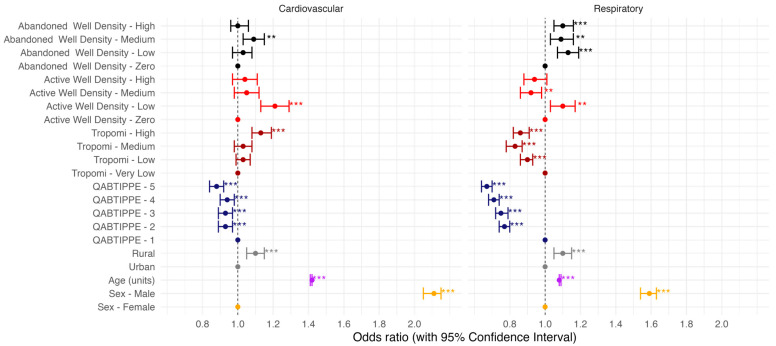
The association between well density (abandoned and active, respectively in black and red), TROPOMI NO2 (in dark red), and cardiovascular- and respiratory-related outcomes in Alberta Canada, adjusted for sex (in orange), age (in purple), location (in grey), and income (in dark blue). QABTIPPE (Neighbourhood Income Quintile Before Tax) was used as a proxy for income. Abandoned and active well densities and TROPOMI NO2 concentrations were classified into four categories (zero/very low to high). * *p* < 0.5; ** *p* < 0.01; *** *p* < 0.001.

The total DAD sample for Alberta consisted of 231,382 individuals with a 40–60% male/female ratio (Figure 4). There was no difference in the age distribution between the DAD and the general population above 30 years of age. Figure 5 and Table 2 (full model) show the association between the exposure of interest (TROPOMI NO_2_ concentrations, abandoned and active well densities) and the health outcomes of interest while adjusting for related outcomes such as sex, age, location (rural/urban), and income. Age, sex male, and living in rural areas were associated with significantly increased odds of cardiovascular and respiratory related outcomes, while higher income was associated with lower risk of cardiovascular- and respiratory-related outcomes. In Alberta, the energy sector is largely dominated by men, particularly in field positions [78,79]. Presumably, this means that employment in this sector is probably associated with poor health outcomes [80,81].

In terms of the variables of interest for this study, Figure 5 and Table 2 show that there is a strong trend for TROPOMI NO_2_ and well density to be associated with increased odds of cardiovascular related outcomes. However, only TROPOMI at the highest level of exposure (aOR = 1.13 [1.08–1.19]), active well density at the lowest level of exposure (1.21 [1.13–1.29]), and abandoned well density at the medium level (1.09 [1.03–1.15]) were statistically significant.

Active well density at the lowest level of exposure (1.10 [1.03–1.17]) and abandoned well densities at low (1.13 [1.07–1.19]), medium (1.09 [1.07–1.16]), and high (1.10 [1.05–1.16]) exposure levels were also associated with increased odds of respiratory-related outcomes, but for TROPOMI NO_2_ the opposite was true for all three levels of exposure with decrease odds (0.83, 0.86, and 0.90) of respiratory-related outcomes (Table 2; all models tested in Tables S3 and S4).

Our study suggests that, like in most jurisdictions in North America, oil and gas activities in Alberta are associated with negative impacts on human health. On average, our results indicate that there is a 9–21% increase in the odds of cardiovascular and respiratory outcomes with various level of exposure to well density, which, in terms of severity, is in line with similar studies dealing with a large population being exposed [82]. However, the odds of having health issues may be higher for people living near wells [7,32]. Previous U.S. studies have shown a link between poorer cardiovascular health and living near oil and gas sites [32,83]. Similarly, living in proximity to oil and gas infrastructure can result in poor respiratory outcomes [7,84,85].

There is reasonably good evidence that increasing exposure to NO_2_ is associated with a higher likelihood of cardiovascular and respiratory morbidity [86,87]. The results of our analysis are consistent with prevailing evidence for cardiovascular morbidity but are contradictory for respiratory morbidity. We hypothesize that this may reflect residual confounding, such as smoking, inactivity, or, more likely, the effects of other pollutants such as VOCs or PM_2.5_ that could not be included in our models [88]. For example, one study found a negative and significant association between ozone and lung cancer, but this association became insignificant when NO_2_ was included in the model, highlighting the importance of including multiple pollutants when possible [89], as well as considering that there might be synergistic or antagonistic health effects of combined pollutants [90].

Nitrogen oxides (~500 kt in 2022) are the most frequently emitted pollutants in Alberta, followed by VOCs (~450 kt in 2022) and PM_2.5_ (~300 kt in 2022). Total emitted PM_2.5_ and VOCs could not be included in our model because spatial (e.g., satellite measurements) and provincial data were not available for the period of interest. Among the top three emitted pollutants, only NO_x_ (~40%) and VOCs (~30%) have the oil and gas industry as one of their main sources. Nitrogen oxides also did not necessarily correlate well with PM_2.5_ [91]. For example, TROPOMI NO_2_ was much higher in the Edmonton and Calgary areas, where there is heavy traffic, and north of Fort McMurray, where there are large oil sand facilities (Figure S7). This spatial distribution of TROPOMI NO_2_ in Alberta was similar to what was observed by Copper et al. between July 2018 and June 2019 [92]. Compared to PM_2.5_, the highest concentrations measured for the same year were found north of Calgary (Figure S8). The major sources of PM_2.5_ in Alberta were most often found outside large urban areas with dust and fires (which can fluctuate from year to year), followed by agriculture, with these factors being the largest contributors to air pollution. It is possible that other aerosols such as PM_2.5_ (due its small size) were more dangerous in their ability to penetrate deeper into the respiratory system, while NO_2_ may have a more important role in cardiovascular diseases. In addition to that, it is known for Alberta that smoking is more prevalent among men, more so among Canadian-born people than immigrants, and that the smoking rate tends to increase with rurality [93]. Rural and remote areas in Alberta also have a lower life expectancy and more chronic conditions than urban and metro areas [94].

### 3.7. Limitations

It is important to acknowledge some of the main limitations of this study. Our emissions estimates and the federal emissions estimates of VOCs, PM_2.5_, NO_x_ are based on activity data and emission factors. Measurement studies for most of these pollutants emitted by the oil and gas are limited in Canada, but this is also true for most oil and gas-producing countries. Therefore, we recommend continuous emissions monitoring of these pollutants at active and inactive sites to provide a more complete picture of local and regional exposure to pollutant emissions emitted by the upstream oil and gas sector. In combination with tracers, these measurements could also help to (1) determine more accurately the percentage of pollutant emissions produced by upstream oil and gas activities and (2) identify the type of activity contributing the most to these emissions. Second, our assumption that populations are uniformly distributed within each DA might have led to the overestimation or underestimation of the distance between each individual and wells located inside each DA, particularly in rural areas with large dissemination areas. And last, it is hard to know how mobility may have had an impact as the model assumes that people live and work in the same area and that no one moves.

### 3.8. Extensibility to Other Jurisdictions

This study offers valuable insights that could inform assessments in other midsized oil and gas-producing nations, especially where data on emissions and health impacts are limited. Key aspects likely applicable include the following:

− *Emissions Profiles and Trends*: Similar nations may exhibit comparable emissions patterns, with flaring, venting, and fugitive emissions contributing significantly to methane, VOC, and NOx levels. Trends such as stable onsite fuel use but increasing flaring and venting due to regulatory shifts could also have impacts.
− *Geographical Distribution of Emissions*: The clustering of pollutant emissions around oil-producing regions, often in rural areas, is likely a common feature. This could lead to uneven population exposure, particularly affecting rural and Indigenous communities or other socioeconomically marginalized groups.
− *Health Impacts*: The observed link between proximity to oil and gas activities and increased odds of cardio-respiratory health outcomes underscores a pattern that could emerge in other regions with significant oil and gas infrastructure. This aligns with global studies showing similar health risks, suggesting that localized data could reveal comparable health disparities. The lack of agreement for respiratory morbidity in our study was possibly due confounding factors such as smoking, which was more prevalent in rural than urban areas, or other important pollutant/aerosols (VOCs, PM_2.5_) not included in the models.
− *Data Gaps and Policy Challenges*: Like Canada, many midsized producers rely on aggregated emissions inventories or outdated factors, making accurate quantification difficult. This study highlights the importance of combining satellite-based measurements with ground data to improve accuracy and track regulatory impact.
− *Regulatory Implications*: Our findings stress the need for targeted policies in these nations to address emissions and protect vulnerable populations. Tailored interventions could include stricter flaring and venting regulations, enhanced monitoring, and health-focused risk assessments to inform public health strategies. Efforts aligned with these objectives are being carried out by the United Nations Environment Programme and the World Bank, and they must continue.

By applying methodologies from this study, such as leveraging satellite data and conducting regional health analyses, other nations could better quantify their emissions, understand exposure risks, and develop effective mitigation strategies.

## 4. Conclusions

This study investigated the environmental and health impacts of emissions from oil and gas activities in Alberta, focusing on flaring, venting, fugitive emissions, and onsite fuel use. This is also the first study in Canada to map the spatial distributions of some major pollutants emitted by upstream oil and gas activities, assess the sociodemographic profile of the population living near active oil and gas infrastructure, and characterize the association between exposure to oil and gas sites and both cardiovascular and respiratory outcomes.

Alberta, a significant contributor to Canada’s methane, VOC, and NOx emissions, exhibits spatial disparities in pollutant concentrations, with hotspots near oil-rich areas like Lloydminster and Grande Prairie. Approximately 9% of Alberta’s population lives within 1 km of active wells, with rural and Indigenous populations disproportionately affected. Health analyses revealed associations between exposure to well proximity/density and increased odds of cardiovascular and respiratory outcomes, mirroring findings from U.S. studies.

Despite this initial work, the association between exposure to NO_x_, VOC, and PM_2.5_ emissions from upstream oil and gas operations and health outcomes in Alberta is still under-studied. Future studies should include emissions from all oil and gas sources combined with field measurements to better assess the influence of oil and gas on health-related issues.

## Figures and Tables

**Table 1:** A summary of the sociodemographic variables of Alberta based on the 2016 Canadian Census. For some of these variables (e.g., visible minority), the total population from the short questionnaire may differ slightly than the total population derived from the 25% sample data (long questionnaire). European is defined here as an individual that did not make up part of a visible minority or Aboriginal identity.

Variables	Alberta
Population, *n*	4,067,175
Census dissemination areas, n	5803
Race/ethnicity, *n* (%)	
European, *n* (% population)	2,875,370 (70.6)
Aboriginal identity, *n* (% population)	258,640 (6.4)
Visible minority, *n* (% population)	933,165 (23.0)
Latin American, *n* (% of Visible minority)	55,090 (5.9)
Black *n*, (% of Visible minority)	129,390 (13.9)
Asian *n*, (% of Visible minority)	487,535 (52.2)
Other *n*, (% of Visible minority)	261,150 (28.0)
Education, *n* (% population aged 15 years and over)	
No diploma	540,775 (16.9)
Secondary high school diploma	895,885 (27.9)
Post-secondary diploma	1,769,500 (55.2)
Renter-occupied households, *n* (% household type)	412,150 (30.0)
Income, *n* (% population aged 15 years and over)	
Under CAD 30,000	1,139,730 (37.4)
CAD 30,000 to CAD 60,000	840,125 (27.5)
CAD 60,000 to CAD 90,000	504,200 (16.5)
Over CAD 90,000	569,420 (18.6)
Age characteristics, n (% population)	
0–4 years	266,515 (6.6)
5 to 9 years	270,715 (6.7)
10 to 14 years	241,920 (5.9)
65 years and over	500,215 (12.3)

**Table 2:** The association between the exposure of interest (TROPOMI NO_2_ concentrations, and abandoned and active well densities) and the health outcomes of interest (cardiovascular and respiratory issues) adjusted for age, sex, income (QABTIPPE; Neighbourhood Income Quintile Before Tax), and location (urban/rural). Urban/rural is defined using the Statistical Area Classification type (SACtype). For this study, SACtype descriptions 1 (census subdivision with census metropolitan area), 2, and 3 are designated as urban and categories 4 to 8 (census subdivision withing territories, outside of census agglomeration) as rural. Bold indicates statistical significance.

Predictors	Cardiovascular	Respiratory
Sex [M]	2.11 [2.05–2.16]	1.59 [1.54–1.63]
Age	1.42 [1.41–1.42]	1.08 [1.08–1.09]
Location [rural]	1.10 [1.04–1.15]	1.44 [1.37–1.51]
Income		
QABTIPPE [2]	0.93 [0.89–0.97]	0.77 [0.74–0.80]
QABTIPPE [3]	0.93 [0.89–0.97]	0.75 [0.72–0.79]
QABTIPPE [4]	0.94 [0.90–0.98]	0.71 [0.68–0.74]
QABTIPPE [5]	0.88 [0.84–0.92]	0.67 [0.64–0.70]
TROPOMI NO_2_		
Low	1.03 [0.99–1.07]	**0.90 [0.86–0.93]**
Medium	1.03 [0.98–1.08]	**0.83 [0.78–0.87]**
High	**1.13 [1.08–1.19]**	**0.86 [0.82–0.91]**
Active Well Density		
Low	**1.21 [1.13–1.29]**	**1.10 [1.03–1.17]**
Medium	1.05 [0.98–1.12]	0.92 [0.86–0.98]
High	1.04 [0.97–1.11]	0.94 [0.88–1.01]
Abandoned Well Density		
Low	1.03 [0.97–1.08]	**1.13 [1.07–1.19]**
Medium	**1.09 [1.03–1.15]**	**1.09 [1.07–1.16]**
High	1.00 [0.96–1.06]	**1.10 [1.05–1.16]**

## Data Availability

Oil and gas volumetric data used for this study are available on the Petrinex data portal (https://www.petrinex.ca/PD/Pages/APD.aspx, accessed on 23 May 2024). The data from the Alberta Airsheds (https://www.alberta.ca/access-air-data, accessed on 23 May 2024) and ECCC (https://data-donnees.az.ec.gc.ca/data/air/monitor/national-air-pollution-surveillance-naps-program/, accessed on 6 June 2023) field monitoring stations are also publicly available. Only approved researchers can have access to the CanCHEC dataset at the research data centres.

## Supplementary Materials

The following supporting information can be downloaded at https://www.mdpi.com/article/10.3390/ijerph21121692/s1, File S1: Activity and CH_4_ emission factors; File S2: Pollutant Inventory equations; Figure S1: Active facility and well geolocations for inventoried years (2016 to 2021) in Alberta. Field operational areas are also shown; Figure S2: Population density (people per km^2^) of Alberta based on the 2016 Canadian Census. Each polygon represents a dissemination area. Zoom in (25-km radius) on the two largest cities, Edmonton and Calgary, of Alberta. Figure S3: Exposure to VOCs and NOx for selected municipalities (n = 72) in Alberta. Proportion of people affected is defined as the number of people within 1-km of active wells divided by the total population of the municipality. Municipalities with a population below 500 (consisting mainly of summer villages) and above 600,000 (Calgary and Edmonton don’t have oil and gas well inside the city limits) were excluded and log scale was used for better visualization. The size of the circles is proportional to the estimated population. Circles are colored by estimated pollutant emissions. Figure S4: Relationship between population size and annual (2021) NO_x_ mean concentrations (ppb) for monitoring stations (n = 86) from the ECCC National Air Pollution Surveillance Program. Municipalities with a population above 200,000 were excluded for better visualization. Figure S5: Relationship between mean annual NO_x_ (ppb) measured at airshed monitoring stations and TROPOMI tropospheric vertical column densities of NO_2_ in Alberta during 2021. The colored horizontal bands are based (NO_x_ concentration) on the Canadian Ambient Air Quality Standards (CAAQS) and management levels (red, orange, yellow, and green). Figure S6: Airsheds field monitoring stations (n = 70) across Alberta. Statistics Canada dissemination areas (polygons) are also shown (n = 5803). Zoom in (25-km radius) on the two largest cities, Edmonton and Calgary, of Alberta. Figure S7. TROPOMI tropospheric vertical column densities of NO_2_ in Alberta during 2021. Zoom in (25-km radius) with city limits on Fort McMurray, Edmonton, Red Deer and Calgary. Figure S8. Satellite-derived surface PM_2.5_ concentrations over Alberta during 2021. Surface fine particulate matter (PM2.5) estimates are from the V5.GL.03 dataset. The V5.GL.03 surface PM_2.5_ concentrations are estimated by combining aerosol optical depth (AOD) values from several satellite instruments (NASA MODIS, MISR, and SeaWiFS) and the GEOS-Chem chemical transport model. The combined AOD values are related to surface PM_2.5_ using the AOD to PM_2.5_ relationships simulated with GEOS-Chem. The final PM_2.5_ estimates are then calibrated to global ground measurements using a Geographically Weight Regression model. The PM_2.5_ estimates are provided at monthly and annual grids with a 1 × 1 km^2^ pixel resolution. Table S1: Active well counts for inventoried years in Alberta. Table S2: Active facility counts for inventoried years in Alberta. Table S3. Association between the exposure of interest (TROPOMI NO_2_ concentrations, and abandoned and active well densities) and the health outcomes of interest (**respiratory** issues) adjusted for age, sex, income (QABTIPPE; Neighborhood Income Quintile Before Tax), and location (urban/rural). Urban/Rural is defined using the Statistical Area Classification type (SACtype). For this study, SACtype description 1 (census subdivision with census metropolitan area), 2, and 3 are designated as urban, and categories 4 to 8 (census subdivision withing territories, outside of census agglomeration) as rural. Table S4. Association between the exposure of interest (TROPOMI NO_2_ concentrations, and abandoned and active well densities) and the health outcomes of interest (**cardiovascular** issues) adjusted for age, sex, income (QABTIPPE; Neighborhood Income Quintile Before Tax), and location (urban/rural). Urban/Rural is defined using the Statistical Area Classification type (SACtype). For this study, SACtype description 1 (census subdivision with census metropolitan area), 2, and 3 are designated as urban, and categories 4 to 8 (census subdivision withing territories, outside of census agglomeration) as rural.
