# Supplementary material for: Sociodemographic and Population Exposure to Upstream Oil and Gas Operations in Canada
Source: Int J Environ Res Public Health. 2024 Dec 19;21(12):1692. doi: 10.3390/ijerph21121692 (PMC11675219; doi:10.3390/ijerph21121692)
Supplement: Supplementary file 1 [file ijerph-21-01692-s001.zip › ijerph-3341977-supplementary.pdf]

Supplemental Information

Sociodemographic and Population Exposure to Upstream Oil and Gas Operations in Canada

Martin Lavoie [1,*], David Risk [1] and Daniel Rainham [2,3]

[1] Department of Earth and Environmental Sciences, St. Francis Xavier University, Antigonish, NS B2G 2W5, Canada; drisk@stfx.ca
[2] School of Health and Human Performance, Dalhousie University, Halifax, NS B3H 4R2, Canada; daniel.rainham@dal.ca
[3] Healthy Populations Institute, Dalhousie University, Halifax, NS B3H 4R2, Canada
* Correspondence: mlavoie@stfx.ca or martin21skifond@gmail.com

FILE S1. ACTIVITY AND CH_4 EMISSION FACTORS 2

FILE S2. POLLUTANT INVENTORY 3

VOCS 3
$PM_{2.5}$ 4
NO_X 5

FIGURES 6

TABLES 14

File S1: Activity and CH_4 Emission Factors

Source	Activity Data Description (unit)	Emissions factors (kg/unit)
Oil production	Total crude production (10^3 m^3)	1022.16
Gas production	Total gas production (10^6 m^3)	436.89
Venting (oil)	Total crude production (10^3 m^3)	2573.69
Venting (gas)	Total gas production (10^6 m^3)	1431.11
Flaring (oil)	Associated gas flared (10^6 m^3)	10641.85
Flaring (gas)	Non-associated gas flared (10^6 m^3)	13613.88

Source: National Inventory report 1990–2020: Greenhouse gas sources and sinks in Canada.

Canada's submission to the United Nations Framework Convention on climate change

File S2: Pollutant Inventory Equations

VOCs

Eq. (SI.1)

Venting:

$$\mathbf{Emis_{i,j} = yi,j \cdot Voli \cdot \varrho j}$$

where:

$Emis_{i,j}$ = vented emissions of component j in area i (kt)

$y_{i,j}$ = mole fraction of component j in area i

Vol_i = volume of gas vented in area i (10^3 m^3)

p_j = density of component j at standard conditions (101.325 kPa and 15°C) (kg/m^3)

Eq. (SI.2)

Fuel combustion:

$$ER_{i,j} = EF_{i,j} \times Q_i \times HHV \times (1-CF_i) \times gc$$

where:

$ER_{i,j}$ = emission rate of substance j from source i (t/y).

$EF_{i,j}$ = emission factor for source i (ng/J).

Q_i = fuel consumption by source i during study year (m^3/yr).

HHV = higher heating value of the fuel (MJ/m^3).

CF_i = control factor for a specific control measure or device applied to source i which indicates the fraction by which the emissions are reduced (kg/kg). 0 in the absence of any data.

gc = a constant of proportionality used to convert the results to units of t/y. 10-9 (dimensionless)

$PM_{2.5}$

Eq. (SI.3)

Flaring:

$$\mathbf{EF_{i,j} = ER_j \cdot HHV_i}$$

where:

$EF_{i,j}$ = emission factor for area i and pollutant j (g/m^3)

ER_j = flaring emission rate for pollutant j (g/MJ) = 0.057

HHV_i = higher heating value for area i (MJ/m^3)

Eq. (SI.4)

Fuel combustion:

$$\boldsymbol{ER_{i,j} = EF_{i,j} \times Q_i \times HHV \times (1 - CF_i) \times gc}$$

where:

$ER_{i,j}$ = emission rate of substance j from source i (t/y).

$EF_{i,j}$ = emission factor for source i (ng/J).

Q_i = fuel consumption by source i during study year (m^3/yr).

HHV = higher heating value of the fuel (MJ/m^3).

CF_i = control factor for a specific control measure or device applied to source i which indicates the fraction by which the emissions are reduced (kg/kg). 0 in the absence of any data.

gc = a constant of proportionality used to convert the results to units of t/y. 10-9 (dimensionless)

NO_X

Eq. (SI.5)

Flaring:

$$EF_{i,j} = ER_j \cdot HHV_i$$

where:

$EF_{i,j}$ = emission factor for area i and pollutant j (g/m^3)

ER_j = flaring emission rate for pollutant j (g/MJ) = 0.0292

HHV_i = higher heating value for area i (MJ/m^3)

Eq. (SI.6)

Fuel combustion:

$$ER_{i,j} = EF_{i,j} \times Q_i \times HHV \times (1 - CF_i) \times gc$$

where:

$ER_{i,j}$ = emission rate of substance j from source i (t/y).

$EF_{i,j}$ = emission factor for source i (ng/J).

Q_i = fuel consumption by source i during study year (m^3/yr).

HHV = higher heating value of the fuel (MJ/m^3).

CF_i = control factor for a specific control measure or device applied to source i which indicates the fraction by which the emissions are reduced (kg/kg). 0 in the absence of any data.

gc = a constant of proportionality used to convert the results to units of t/y. 10-9 (dimensionless)

Figures

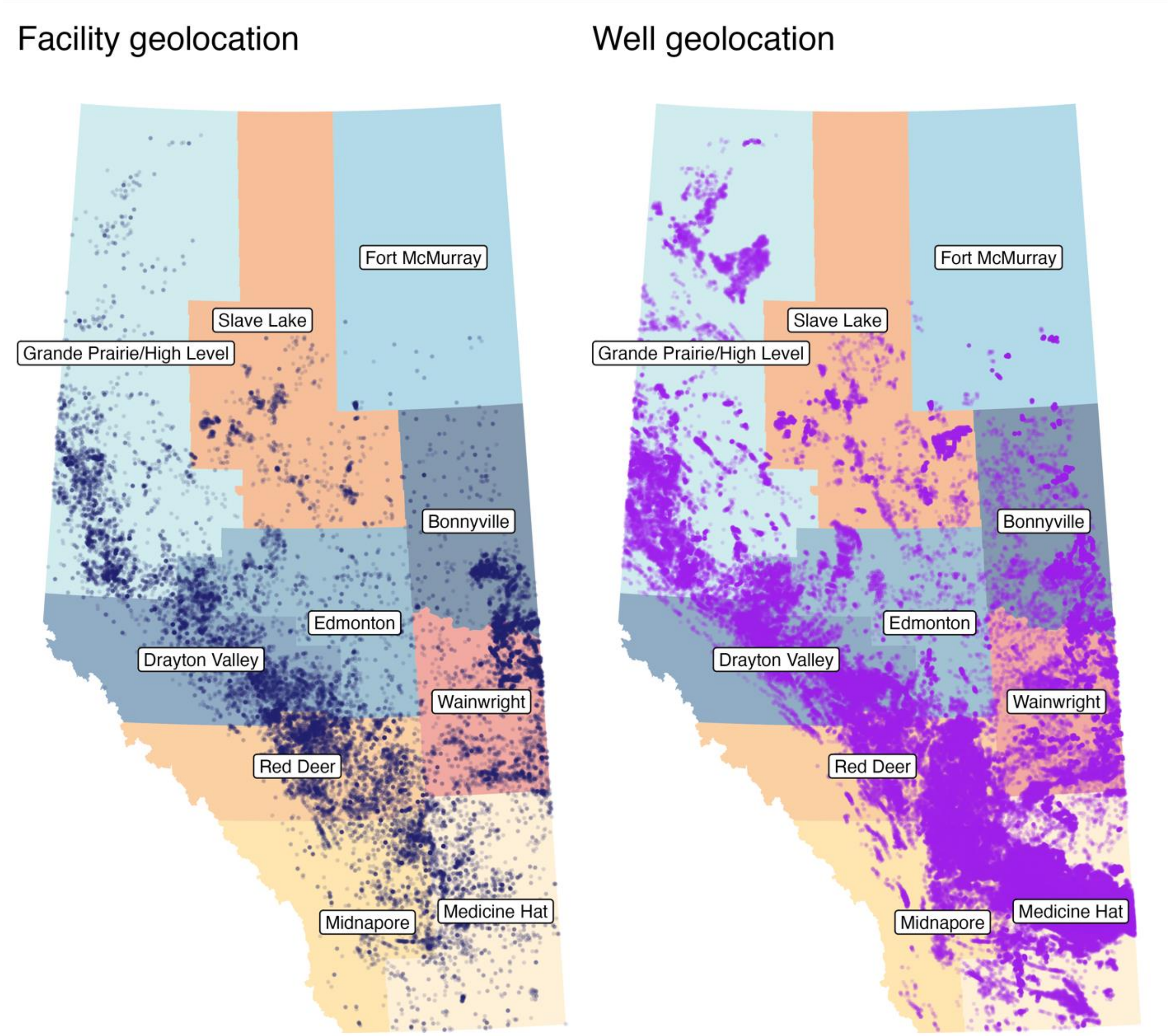

Figure S1: Active facility and well geolocations for inventoried years (2016 to 2021) in Alberta. Field operational areas are also shown.

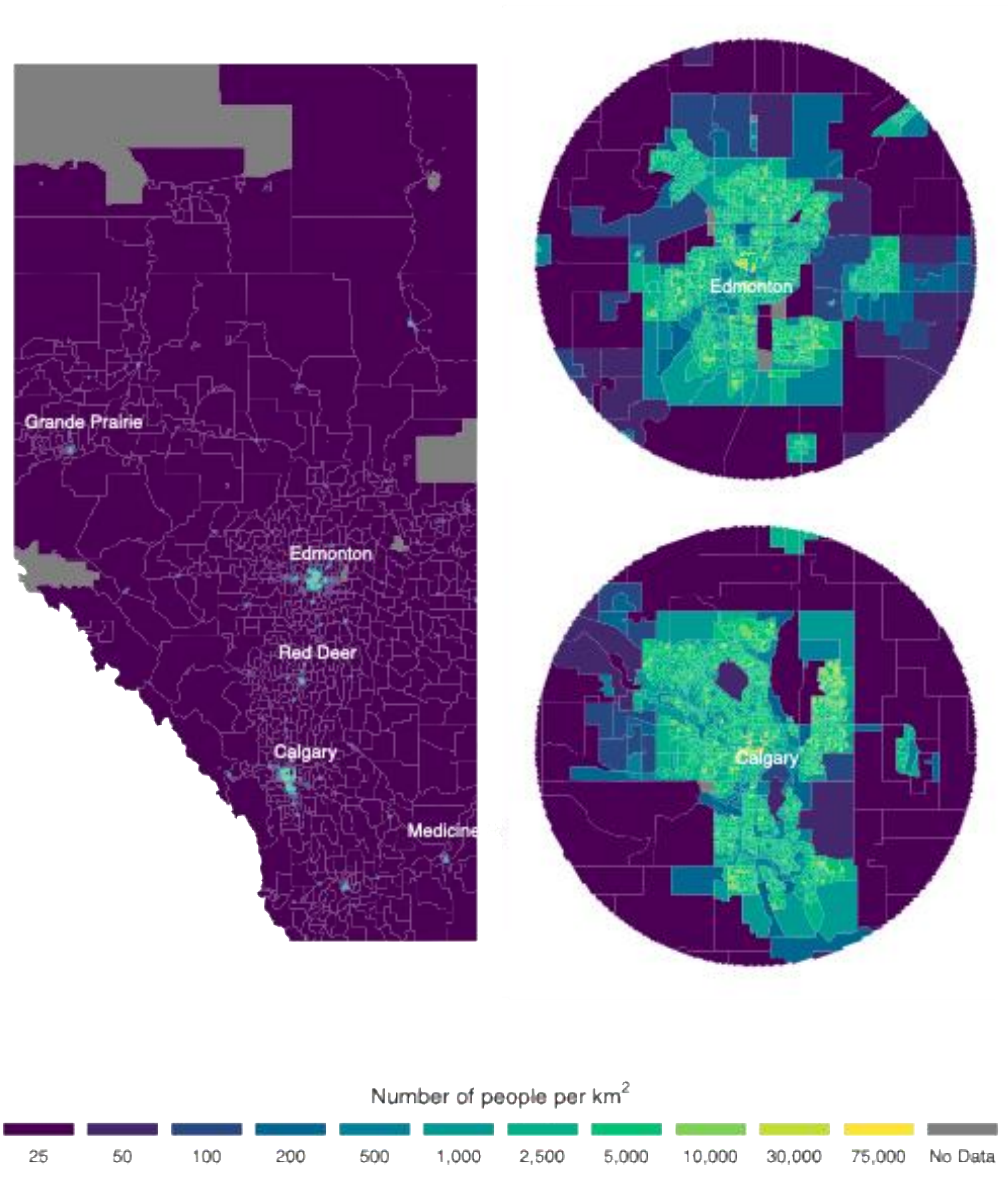

Figure S2: Population density (people per km²) of Alberta based on the 2016 Canadian Census. Each polygon represents a dissemination area. Zoom in (25-km radius) on the two largest cities, Edmonton and Calgary, of Alberta.

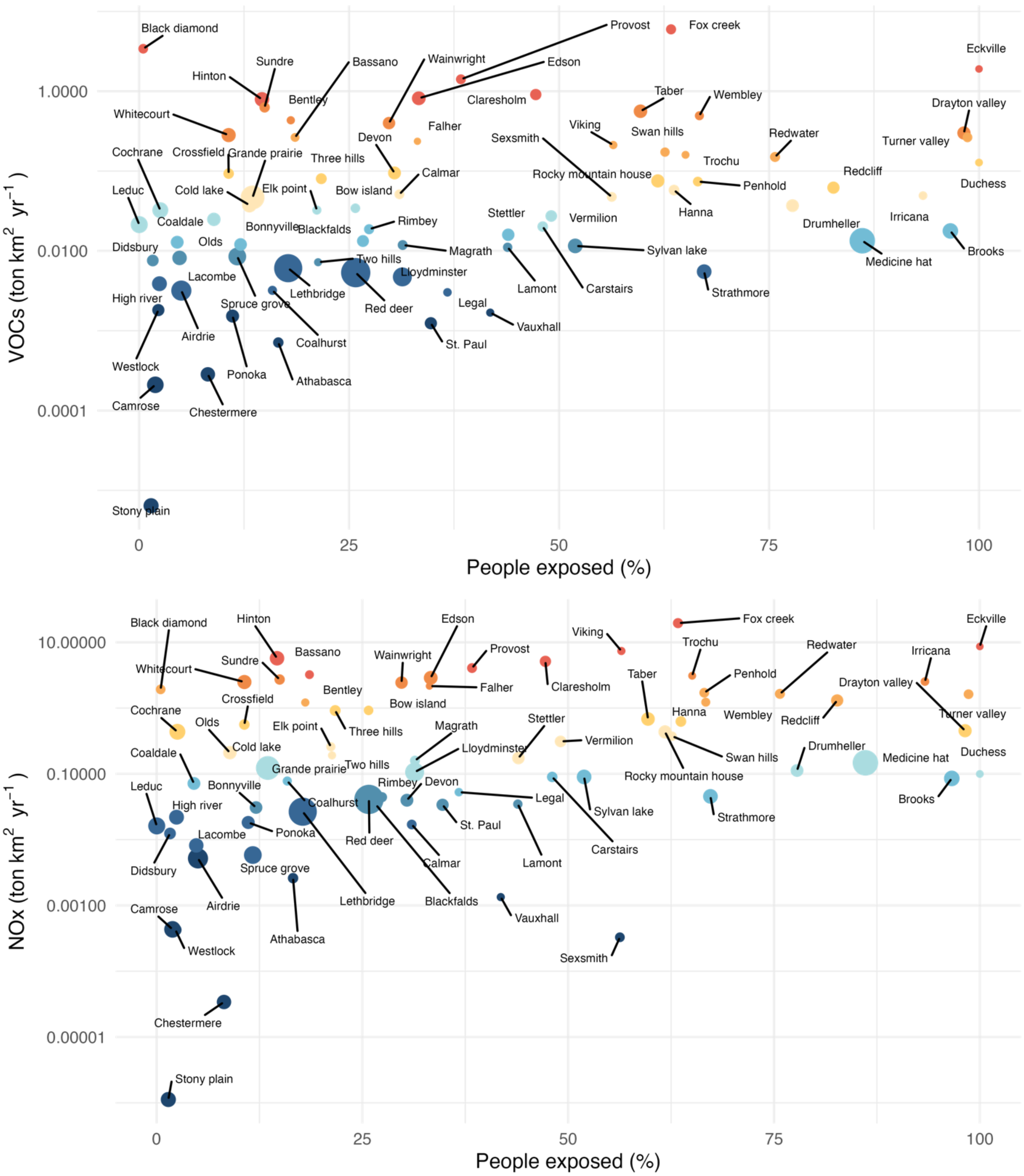

Figure S3: Exposure to VOCs and NOx for selected municipalities (n = 72) in Alberta. Proportion of people affected is defined as the number of people within 1-km of active wells divided by the total population of the municipality. Municipalities with a population below 500 and above 600,000 were excluded and log scale was used for better visualization. The size of the circles is proportional to the estimated population. Circles are colored by estimated pollutant emissions.

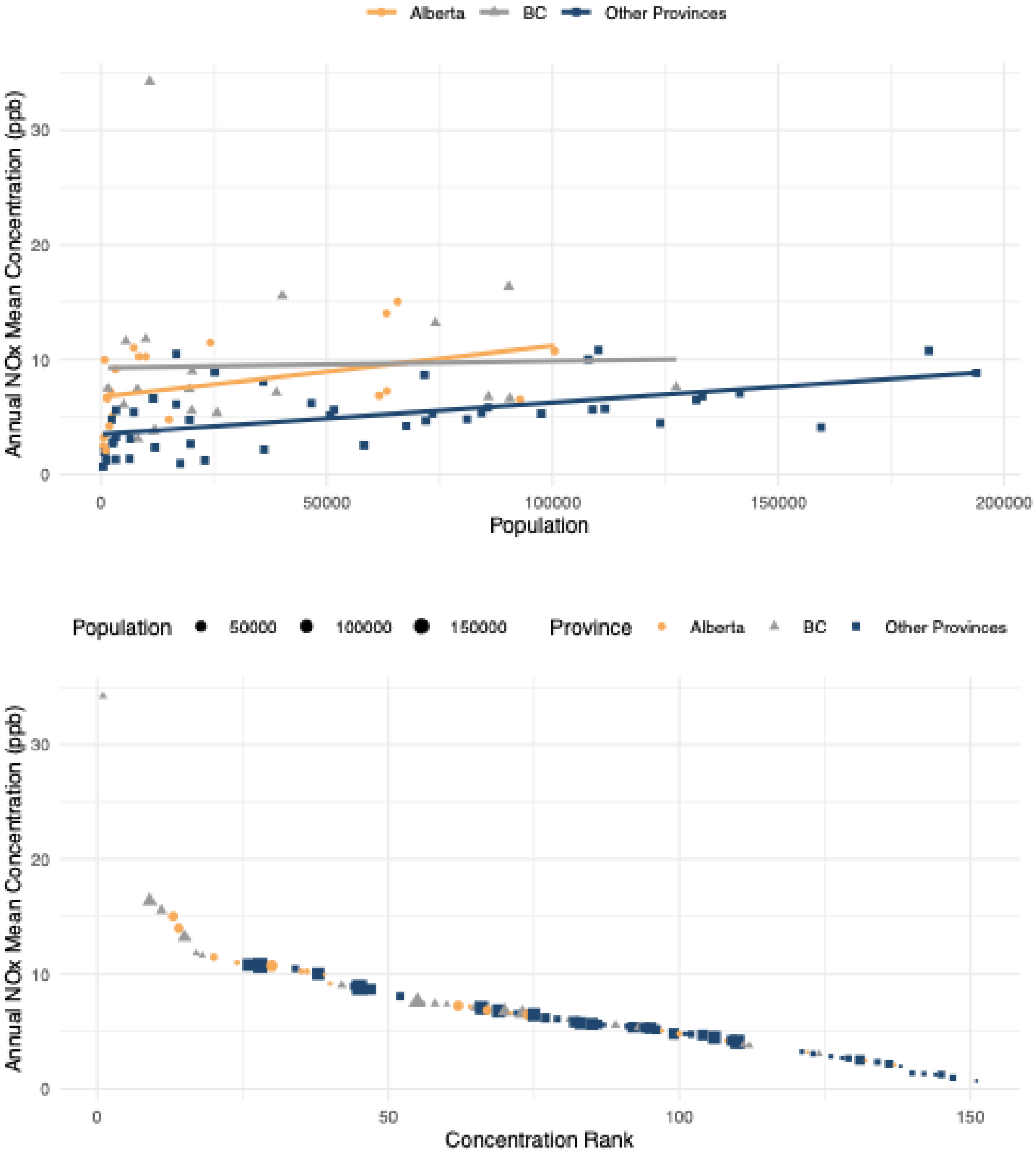

Figure S4: Relationship between population size and annual (2021) NO_x mean concentrations (ppb) for monitoring stations (n = 86) from the ECCC National Air Pollution Surveillance Program. Municipalities with a population above 200,000 were excluded for better visualization.

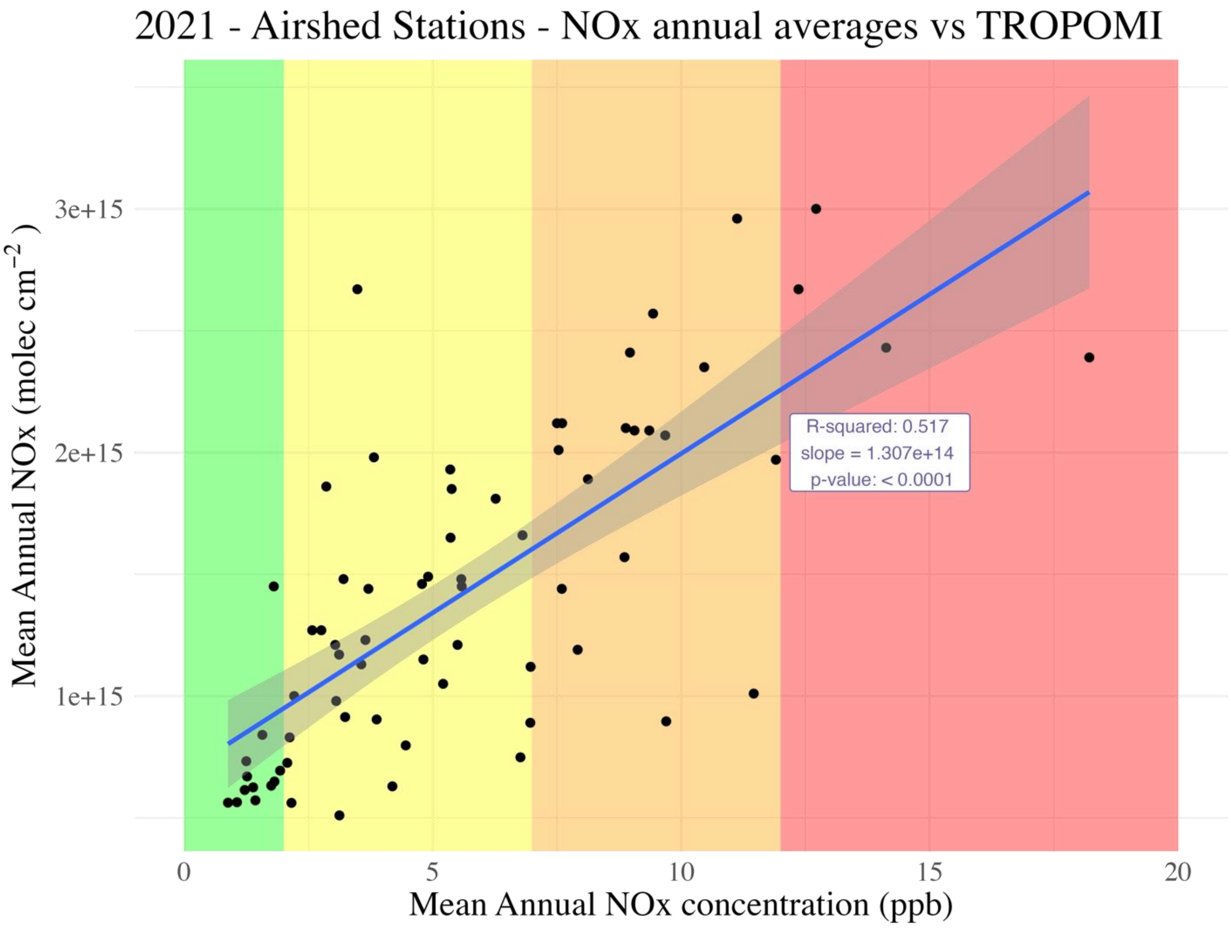

Figure S5: Relationship between mean annual NO_x (ppb) measured at airshed monitoring stations and TROPOMI tropospheric vertical column densities of NO_2 in Alberta during 2021. The colored horizontal bands are based (NO_x concentration) on the Canadian Ambient Air Quality Standards (CAAQS) and management levels (red, orange, yellow, and green).

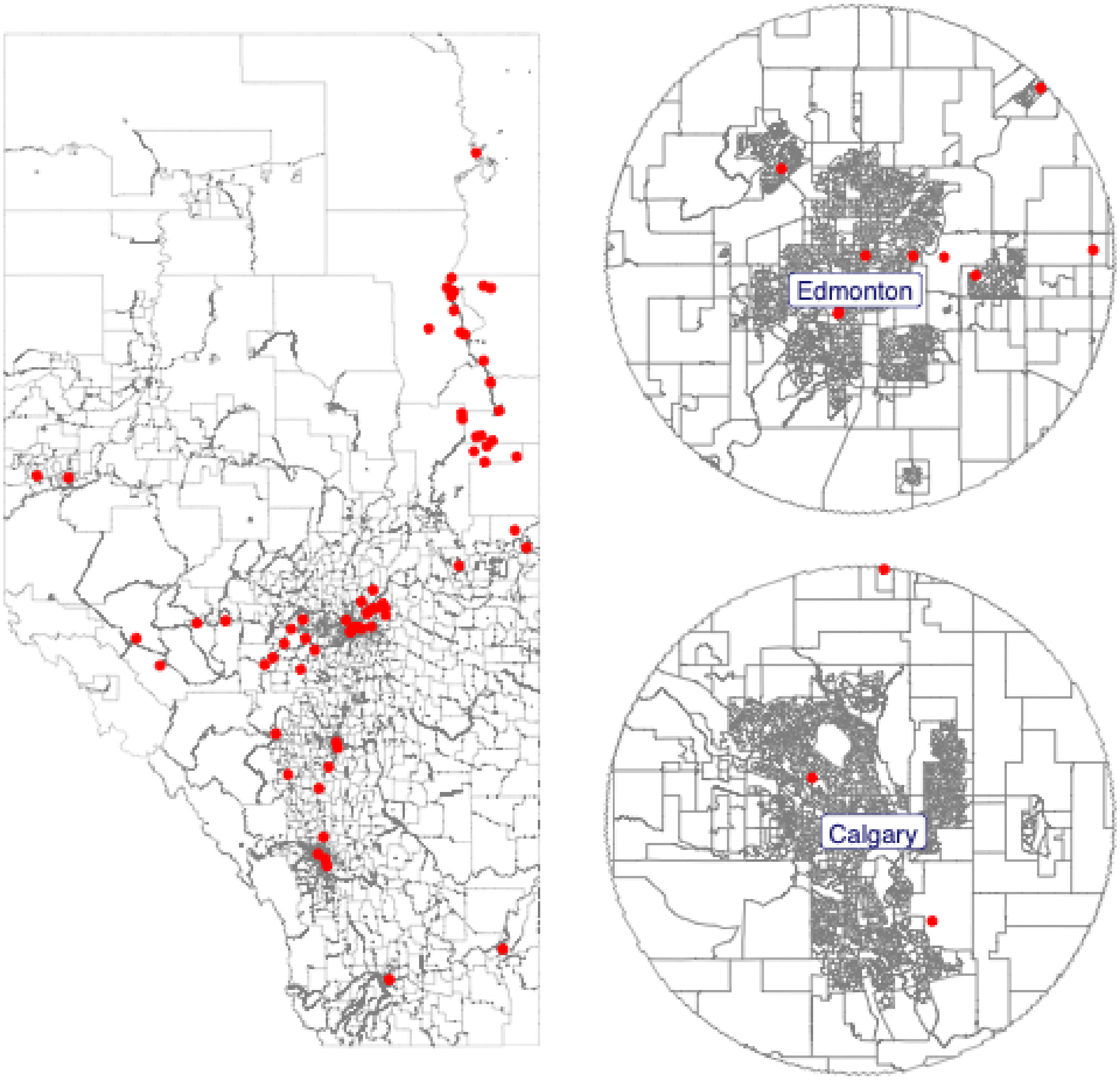

Figure S6: Airsheds field monitoring stations (n = 70) across Alberta. Statistics Canada dissemination areas (polygons) are also shown (n = 5803). Zoom in (25-km radius) on the two largest cities, Edmonton and Calgary, of Alberta.

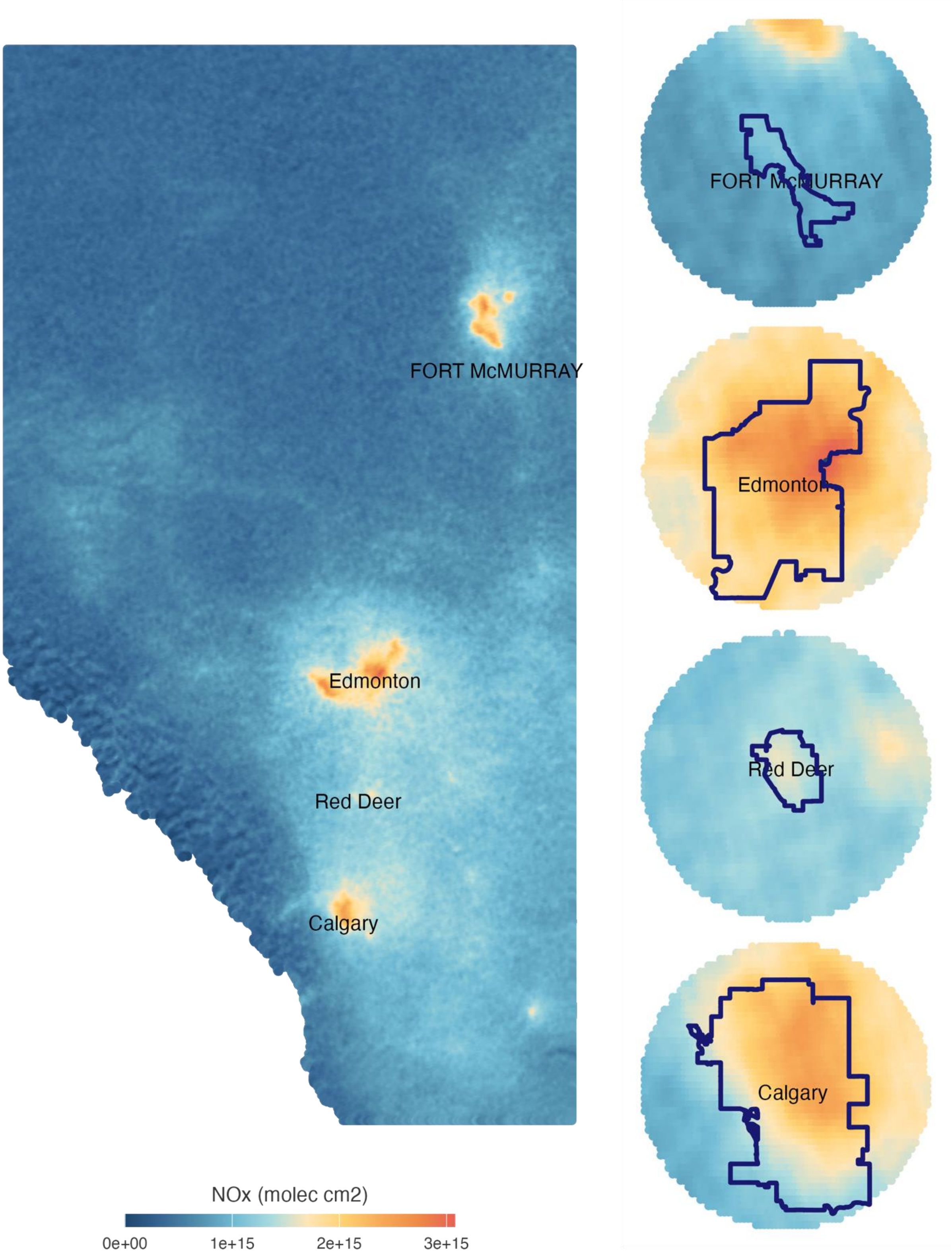

Figure S7. TROPOMI tropospheric vertical column densities of NO_2 in Alberta during 2021. Zoom in (25-km radius) with city limits on Fort McMurray, Edmonton, Red Deer and Calgary.

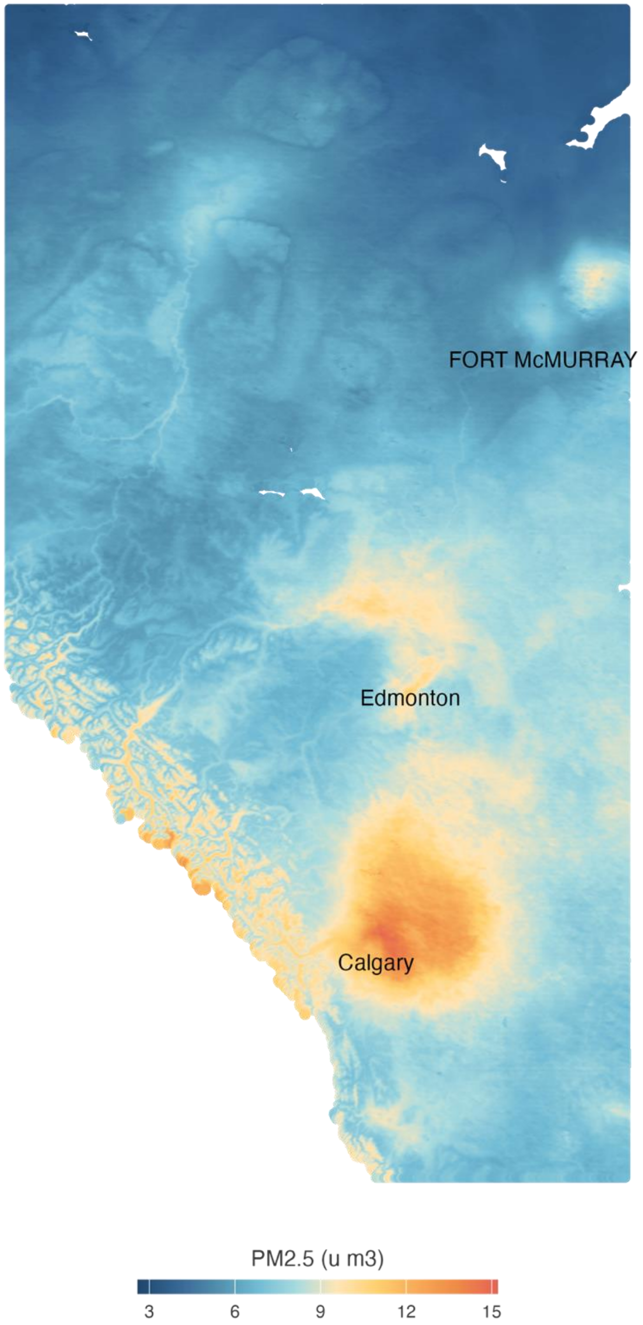

Figure S8. Satellite-derived surface $PM_{2.5}$ concentrations over Alberta during 2021. Surface fine particulate matter (PM2.5) estimates are from the V5.GL.03 dataset. The V5.GL.03 surface $PM_{2.5}$ concentrations are estimated by combining aerosol optical depth (AOD) values from several satellite instruments (NASA MODIS, MISR, and SeaWiFS) and the GEOS-Chem chemical transport model. The combined AOD values are related to surface $PM_{2.5}$ using the AOD to $PM_{2.5}$ relationships simulated with GEOS-Chem. The final $PM_{2.5}$ estimates are then calibrated to global ground measurements using a Geographically Weight Regression model. The $PM_{2.5}$ estimates are provided at monthly and annual grids with a 1 x 1 km^2 pixel resolution.

Tables

Table S1: Active well counts for inventoried years in Alberta.

Well Type	2016	2017	2018	2019	2020	2021
Bitumen	12,760	12,677	12,336	11,457	10,669	10,349
Coalbed methane	21,694	21,633	21,464	21,285	20,848	20,911
Crude Oil	34,730	34,368	34,575	33,570	31,843	31,385
Gas	97,649	95,766	94,229	87,964	84,430	83,501
Water	426	387	373	320	252	184
**Total**	167,259	164,831	162,977	154,596	148,042	146,330

Table S2: Active facility counts for inventoried years in Alberta.

Facility Type	2016	2017	2018	2019	2020	2021
Bitumen Battery	2,743	2,703	2,625	2,414	2,137	1,999
Gas Battery	9,701	9,207	8,886	7,962	7,329	7,052
Gas Gathering System	2,865	2,760	2,740	2,575	2,407	2,321
Gas Processing Plant	549	525	512	479	460	441
Injection Plant	149	154	176	164	150	167
Oil Battery	7,920	7,797	7,697	7,279	6,672	6,594
**Total**	23,927	23,147	22,636	20,873	19,155	18,574

Table S3. Association between the exposure of interest (TROPOMI NO_2 concentrations, and abandoned and active well densities) and the health outcomes of interest (**respiratory** issues) adjusted for age, sex, income (QABTIPPE; Neighborhood Income Quintile Before Tax), and location (urban/rural). Urban/Rural is defined using the Statistical Area Classification type (SACtype). For this study, SACtype description 1 (census subdivision with census metropolitan area), 2, and 3 are designated as urban, and categories 4 to 8 (census subdivision withing territories, outside of census agglomeration) as rural.

	**Base Model**		**Model 1**		**Model 2**		**Model 3**		**Model 4**		**Model 5**	
**Predictors**	*Odds Ratios*	*CI*	*Odds Ratios*	*CI*	*Odds Ratios*	*CI*	*Odds Ratios*	*CI*	*Odds Ratios*	*CI*	*Odds Ratios*	*CI*
(Intercept)	0.04 ***	0.04 – 0.04	0.06 ***	0.05 – 0.06	0.08 ***	0.07 – 0.08	0.06 ***	0.05 – 0.06	0.05 ***	0.05 – 0.06	0.05 ***	0.05 – 0.06
GENDER CODE [M]	1.60 ***	1.55 – 1.64	1.61 ***	1.56 – 1.65	1.59 ***	1.55 – 1.64	1.59 ***	1.54 – 1.63	1.59 ***	1.54 – 1.63	1.59 ***	1.54 – 1.63
AGE GROUP	1.08 ***	1.08 – 1.08	1.08 ***	1.08 – 1.09	1.08 ***	1.08 – 1.09	1.08 ***	1.08 – 1.09	1.08 ***	1.08 – 1.09	1.08 ***	1.08 – 1.09
QABTIPPE [2]			0.75 ***	0.72 – 0.78	0.75 ***	0.72 – 0.78	0.77 ***	0.74 – 0.80	0.77 ***	0.74 – 0.80	0.77 ***	0.74 – 0.80
QABTIPPE [3]			0.74 ***	0.71 – 0.77	0.74 ***	0.71 – 0.78	0.75 ***	0.72 – 0.78	0.76 ***	0.73 – 0.79	0.75 ***	0.72 – 0.79
QABTIPPE [4]			0.69 ***	0.66 – 0.72	0.69 ***	0.66 – 0.72	0.70 ***	0.67 – 0.73	0.72 ***	0.69 – 0.75	0.71 ***	0.68 – 0.74
QABTIPPE [5]			0.65 ***	0.63 – 0.68	0.65 ***	0.62 – 0.68	0.67 ***	0.64 – 0.70	0.68 ***	0.65 – 0.71	0.67 ***	0.64 – 0.70
tropomi x nox group [Low]					0.76 ***	0.73 – 0.79	0.89 ***	0.85 – 0.93	0.89 ***	0.85 – 0.93	0.90 ***	0.86 – 0.93
tropomi x nox group [Medium]					0.61 ***	0.59 – 0.64	0.82 ***	0.78 – 0.86	0.83 ***	0.79 – 0.87	0.83 ***	0.78 – 0.87
tropomi x nox group [Higher]					0.63 ***	0.60 – 0.65	0.84 ***	0.80 – 0.89	0.85 ***	0.81 – 0.90	0.86 ***	0.82 – 0.91
rural [1]							1.51 ***	1.45 – 1.58	1.47 ***	1.40 – 1.54	1.44 ***	1.37 – 1.51
count density active group [Low]									1.18 ***	1.12 – 1.25	1.10 **	1.03 – 1.17
count density active group [Medium]									0.97	0.92 – 1.03	0.92 **	0.86 – 0.98
count density active group [Higher]									1.00	0.95 – 1.07	0.94	0.88 – 1.01
count density aband group [Low]											1.13 ***	1.07 – 1.19
count density aband group [Medium]											1.09 **	1.03 – 1.16
count density aband group [Higher]											1.10 ***	1.05 – 1.16
Observations	231382				231382		231382		231382		231382	
* p<0.05 ** p<0.01 *** p<0.001												

Table S4. Association between the exposure of interest (TROPOMI NO_2 concentrations, and abandoned and active well densities) and the health outcomes of interest (**cardiovascular** issues) adjusted for age, sex, income (QABTIPPE; Neighborhood Income Quintile Before Tax), and location (urban/rural). Urban/Rural is defined using the Statistical Area Classification type (SACtype). For this study, SACtype description 1 (census subdivision with census metropolitan area), 2, and 3 are designated as urban, and categories 4 to 8 (census subdivision withing territories, outside of census agglomeration) as rural.

	**Base Model**		**Model 1**		**Model 2**		**Model 3**		**Model 4**		**Model 5**	
**Predictors**	*Odds Ratios*	*CI*	*Odds Ratios*	*CI*	*Odds Ratios*	*CI*	*Odds Ratios*	*CI*	*Odds Ratios*	*CI*	*Odds Ratios*	*CI*
(Intercept)	0.00 ***	0.00 – 0.00	0.00 ***	0.00 – 0.00	0.00 ***	0.00 – 0.00	0.00 ***	0.00 – 0.00	0.00 ***	0.00 – 0.00	0.00 ***	0.00 – 0.00
GENDER CODE [M]	2.11 ***	2.05 – 2.16	2.11 ***	2.06 – 2.17	2.11 ***	2.06 – 2.17	2.11 ***	2.05 – 2.17	2.11 ***	2.05 – 2.16	2.11 ***	2.05 – 2.16
AGE GROUP	1.42 ***	1.41 – 1.42	1.42 ***	1.41 – 1.42	1.42 ***	1.41 – 1.42	1.41 ***	1.41 – 1.42	1.42 ***	1.41 – 1.42	1.42 ***	1.41 – 1.42
QABTIPPE [2]			0.93 ***	0.89 – 0.97	0.93 ***	0.89 – 0.97	0.94 **	0.90 – 0.97	0.93 **	0.90 – 0.97	0.93 ***	0.89 – 0.97
QABTIPPE [3]			0.92 ***	0.88 – 0.96	0.93 **	0.89 – 0.97	0.93 ***	0.89 – 0.97	0.93 ***	0.89 – 0.97	0.93 ***	0.89 – 0.97
QABTIPPE [4]			0.92 ***	0.88 – 0.96	0.93 ***	0.89 – 0.97	0.93 **	0.89 – 0.97	0.94 **	0.90 – 0.98	0.94 **	0.90 – 0.98
QABTIPPE [5]			0.85 ***	0.81 – 0.89	0.87 ***	0.83 – 0.91	0.88 ***	0.84 – 0.91	0.88 ***	0.84 – 0.92	0.88 ***	0.84 – 0.92
tropomi x nox group [Low]					0.96 *	0.93 – 1.00	1.03	0.99 – 1.08	1.03	0.99 – 1.08	1.03	0.99 – 1.07
tropomi x nox group [Medium]					0.90 ***	0.86 – 0.93	1.02	0.97 – 1.07	1.03	0.98 – 1.09	1.03	0.98 – 1.08
tropomi x nox group [Higher]					0.98	0.94 – 1.02	1.11 ***	1.06 – 1.16	1.13 ***	1.08 – 1.19	1.13 ***	1.08 – 1.19
rural [1]							1.19 ***	1.14 – 1.24	1.11 ***	1.06 – 1.17	1.10 ***	1.04 – 1.15
count density active group [Low]									1.25 ***	1.18 – 1.33	1.21 ***	1.13 – 1.29
count density active group [Medium]									1.08 *	1.01 – 1.15	1.05	0.98 – 1.12
count density active group [Higher]									1.06 *	1.00 – 1.13	1.04	0.97 – 1.11
count density aband group [Low]											1.03	0.97 – 1.08
count density aband group [Medium]											1.09 **	1.03 – 1.15
count density aband group [Higher]											1.00	0.96 – 1.06
Observations	231382		231382		231382		231382		231382		231382	

* p<0.05 ** p<0.01 *** p<0.001
